# SDH‐deficient renal cell carcinoma: A case report associated with a novel germline mutation

**DOI:** 10.1002/ccr3.4605

**Published:** 2021-10-18

**Authors:** Vassilis Milionis, Dimitrios Goutas, Dimitrios Vlachodimitropoulos, Nikolaos Katsoulas, Iason D. Kyriazis, Evangelos N. Liatsikos, Nikolaos Marinakis, Traeger‐Synodinos Joanne, Andreas C. Lazaris, Nikolaos Goutas

**Affiliations:** ^1^ Istomedica S.A Athens Greece; ^2^ First Department of Pathology School of Medicine The National and Kapodistrian University of Athens–"Laikon" General Hospital of Athens Athenes Greece; ^3^ Laboratory of Forensic Medicine and Toxicology The National and Kapodistrian University of Athens Athens Greece; ^4^ Department of Urology University of Patras Patras Greece; ^5^ Laboratory of Medical Genetics National and Kapodistrian University of Athens St. Sophia Children's Hospital Athens Greece

**Keywords:** nephrology, renal cell carcinoma, succinate dehydrogenase deficiency, urology

## Abstract

The highly syndromic nature of succinate dehydrogenase‐deficient RCCs constitutes their active surveillance and molecular profiling the alpha and omega.

## Graphical abstract

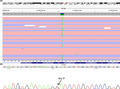

## 1 INTRODUCTION

Routine examination of an asymptomatic 40‐year‐old female patient revealed a right unilateral and unifocal renal mass. The patient underwent a partial nephrectomy, and the renal specimen was sent for histopathologic examination. Molecular testing revealed a heterozygous variant NM_003000.3:c.412G>T, p.(Asp138Tyr) in SDHB gene.

Renal cell carcinoma (RCC) is the most frequent kidney cancer representing over 90% of all renal malignancies.^1^ Histological classification of RCCs is still developing revealing new entities with characteristic morphological features, special immunophenotype, distinctive molecular alterations, or familial predisposition. Among the newest entities are succinate dehydrogenase (SDH)‐deficient renal cell carcinoma (RCC), which was only recently recognized as a distinct subtype in the 2016 World Health Organization classification scheme.^2^ This rare category of renal neoplasms is associated with loss of a mitochondrial enzyme, which participates in both the citric acid cycle and the electron transport chain.

Succinate dehydrogenase (SDH), also known as succinate:ubiquinone oxidoreductase or succinate‐coenzyme Q reductase (SQR) or mitochondrial Complex II, is an enzyme complex localized in the inner mitochondrial membrane which plays an essential role in cellular metabolism regulation by participating in both the Krebs cycle and the electron transport chain. It catalyzes the oxidation of succinate to fumarate in mitochondrial matrix and the reduction of ubiquinone to ubiquinol in the inner mitochondrial membrane by coupling these two reactions.^3^ SDH is a heterotetrameric complex composed of four protein subunits SDHA (flavoprotein), SDHB (iron‐sulfur protein), SDHC (cytochrome), and SDHD (cytochrome). The enzymatic activity of the complex takes place on the hydrophilic head, formed by the SDHA and the SDHB subunits, whereas SDHC and SDHD subunits are hydrophobic membrane anchor subunits, responsible for anchoring the complex to the inner mitochondrial membrane.^3^ There is also another protein known as succinate dehydrogenase assembly factor 2 (SDHAF2) or SDH5 which is necessary for flavinylation and consequently the proper function of SDHA.^4^ Although assembly of SDH subunits occurs at the inner mitochondrial membrane, they are encoded by nuclear autosomal genes [SDHA(5p15.33), SDHB (1p36.13), SDHC(1q23.3), SDHD(11q23), and SDHE(11q12.2)].^5^

Additionally, to its metabolic role in mitochondrial energy generation, SDH has also a role in carcinogenesis as a tumor‐suppressor gene.^6^ Germline mutations in any of the genes encoding SDH subunits have as a result the production of an unstable form of SDH complex and the rapid degradation of SDHB subunit, predisposing to tumorigenesis.^6^ SDH deficiency has been linked with neoplasms such as pheochromocytoma‐paraganglioma, GIST, RCC, and pituitary adenoma in a highly syndromic way.^5^

We report a new case of SDH‐deficient RCC along with a brief review of literature.

## 2 CASE PRESENTATION

**FIGURE 1:**
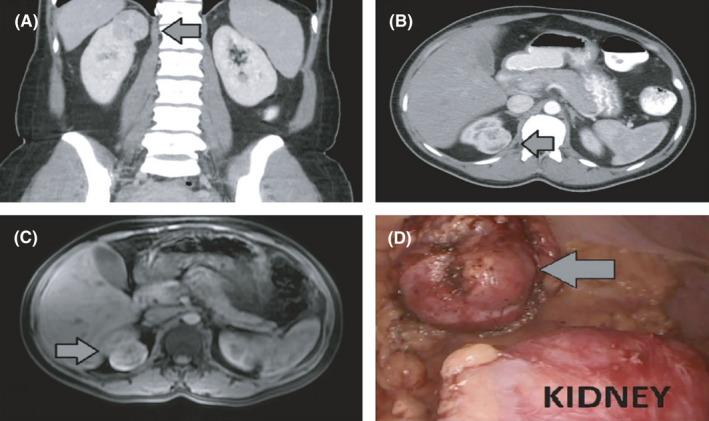
(A‐C) Abdominal computed tomography and magnetic resonance imaging show a large exophytic heterogeneous mass in the upper pole of the right kidney. (D) Intraoperative image of the renal mass

A 40‐year‐old female patient, with no past medical history, presented to the urologic clinic due to an incidental detection of a small renal mass in the upper pole of her right kidney after routine medical examination. She was asymptomatic with no prior urologic history. Her family history was unremarkable. The renal tumor was first identified in a sonographic examination of the upper abdomen and was then further evaluated by computer tomography (CT) and magnetic resonance imaging (MRI). In both CT and MRI, the renal mass was described as a well marginated, heterogeneous mass of 4.8 cm in its maximum dimension (cT1a), which demonstrated heterogeneous contrast enhancement (Figure 1). Patient was subjected to laparoscopic partial nephrectomy. Given the well‐encapsulated mass, a clampless tumor enucleation took place followed by tumor bed renorrhaphy.

**FIGURE 2:**
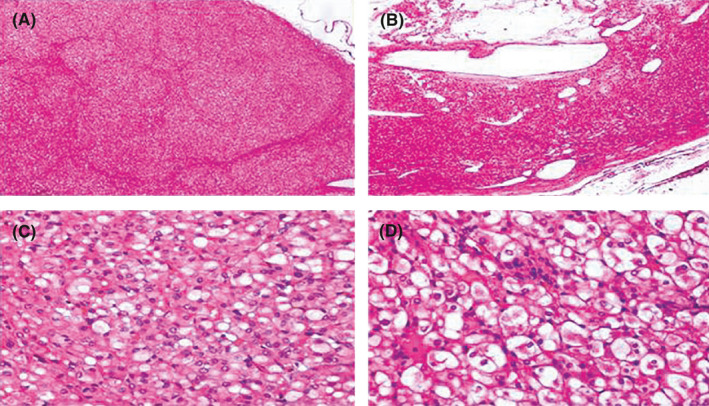
Hematoxylin‐eosin stain **(**A) Vaguely lobular renal tumor (×40) (B) Focal cystic degeneration (×40) (C‐D) Eosinophilic cells with vacuolated cytoplasm and flocculent quality (×400)

Gross examination of the surgical specimen revealed a firm tan brown tumor of 5 cm in its maximum diameter and a few hemorrhagic foci. Histologically, the tumor was well circumscribed, partially encapsulated by a pseudocapsule, with pushing borders and solid or lobular growth pattern with rare foci of cystic degeneration. The neoplastic cells were cuboidal with round to ovoid nuclei. However, there were sites with larger cells and conspicuous nucleoli at ×400 magnification (consistent with an ISUP nucleolar grade 2). The cytoplasm was eosinophilic or flocculent along with readily identified intracytoplasmic vacuoles and inclusion‐like spaces containing eosinophilic often wispy material. Cell borders were indistinct while rare mitotic figures were identified. Entrapment of non‐neoplastic tubules at the periphery of the neoplasm was an additional feature of the neoplasm. There was no necrosis or sarcomatoid change. There was no extrarenal extension (Figure 2).

**FIGURE 3:**
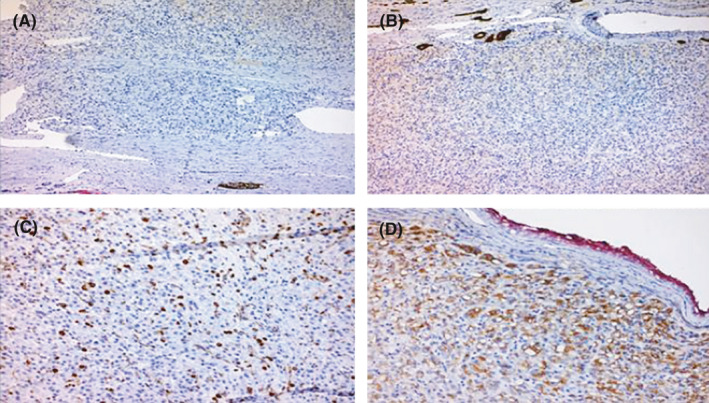
Negative immunostaining expression for (A) CD10 (×100), (B) CK7 (×100), (C) Vimentin with positivity of inflammatory cells (×100), and partially positive expression for (D) EMA (X200)

**FIGURE 4:**
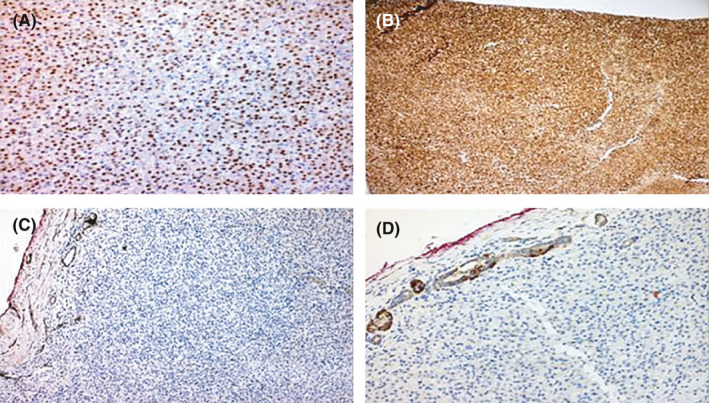
Positive immunostaining expression for (A) PAX8 (×100), (B) SDHA (×100) and (C), (D) negative for SDHB with positivity in renal tubules (×100 and ×200, respectively)

Immunohistochemical (IHC) analysis (Figures 3,4) revealed positive expression for PAX8, EMA, and negative expression for SdhB, Vimentin, CD10, CD117(C‐KIT), CK7, Chromogranin‐A, and Melan‐A. The neoplastic cells were strong positive for SdhA and weak positive for SdhD. Staining for CD117 and Vimentin highlighted any intratumoral inflammatory cells such as mast cells.

12 months after surgical resection our patient did not show any signs of recurrence or metastasis, endorsing the benign course of this type of tumors.

In order to confirm the immunohistocemical results, we further attempted to identify and categorize the exact gene mutation responsible for this neoplastic lesion.

## 3 METHODS

Genetic testing involved semi‐targeted Exome Sequencing using Sophia Genetics Clinical Exome Solution (CES) kit, which includes 4900 genes (114.405 exons). The CES panel includes the genes of interest SDHA, SDHB, SDHC, and SDHD. The patient provided written informed consent for this test. Genomic DNA was extracted from peripheral blood sample via standard procedures using the QiaSympony DNA Robotic system (QIAGEN SA). The resulting CES libraries were sequenced on a NextSeq‐500 (Illumina SA). Bioinformatics analysis was implemented into Sophia DDM platform (Sophia Genetics SA) and VarAFT application.^7^ CES data from the bioinformatic analysis contained 21.259.306 number of reads and 30.263 variants in 4.118 genes. The percentage of regions with at least 25× coverage was 99,56% and the mean coverage was 84×. Variants were classified according to the American College of Medical Genetics and Genomics (ACMG) guidelines.^8^ For variant(s) confirmation targeted Sanger sequencing was performed.

### 3.1 Genetic testing results

**FIGURE 5:**
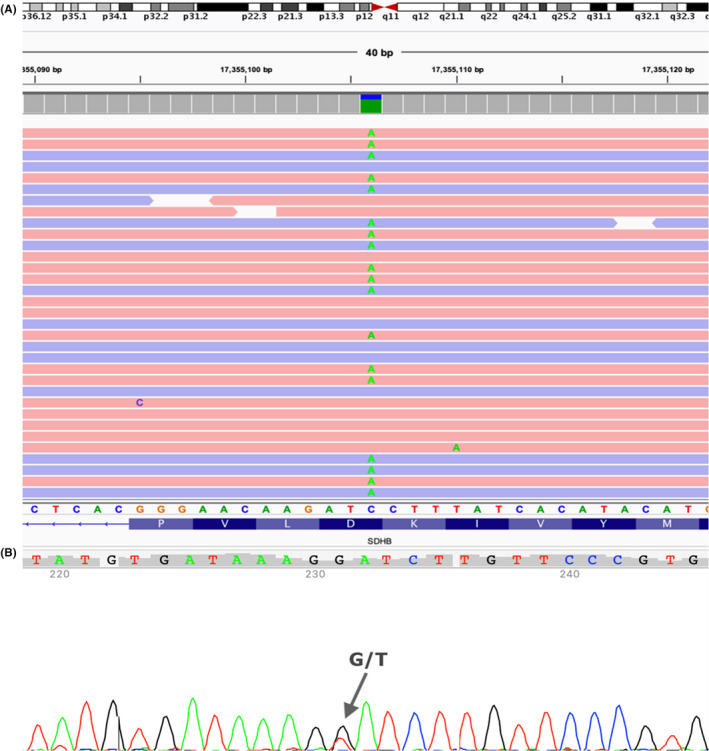
(A) Integrated Genomics Viewer (IGV) screenshot of the SDHB:c.412G > T variant. The variant is shown in reverse strand as C‐to‐A. (B) Sanger Sequencing traces represent the identified variant SDHB:c.412G > T in the proband

Applying filter criteria (phenotype, population frequency, variant type, in‐silico prediction, etc) in CES data, a heterozygous variant NM_003000.3:c.412G>T, p.(Asp138Tyr), in SDHB gene was detected (Figure 5). SDHB gene is associated with non‐syndromic paragangliomas and is inherited with autosomal dominant pattern. The variant c.412G>T was classified according to ACMG guidelines as likely pathogenic (PM2, PM5, PP2, PP3, PP5). This variant has been associated before with the referred condition in ClinVar database (RCV000166877.1); however, it has not been yet related to another SDHB‐deficient RCC. Additionally, a different missense change at the same amino acid residue p.(Asp138Asn) has been determined to be pathogenic.^9^

## 4 DISCUSSION

The metabolic process of citric acid cycle was first described in 1937 by Hans Adolf Krebs^10^ while SDH activity had been, even earlier, detected at 1909 by the Swedish physician Torsten Thunberg.^11^ However, only during the past twenty years, SDH gene mutations have been linked to specific neoplastic and non‐neoplastic human diseases (Table 1).

**TABLE 1:** Neoplastic and non‐neoplastic diseases linked to SDH gene mutations

Neurodegenerative Disorders	Leigh syndrome, leukoencephalopathy, optic atrophy, myopathy, ataxia^12^, ^13^, ^14^, ^15^, ^16^, ^17^
Neoplasms	pheochromocytomas/paraganglioma, GISTs, RCCs, and pituitary adenomas^6^

SDH‐deficient neoplasia refers to all tumors with loss of activity of the mitochondrial complex II. This is almost always a result of a germ line mutation in a gene encoding one of the SDH subunits and a second mutation in wild‐type allele (double‐hit inactivation) causing the whole enzymic complex being non‐functional.^5^ Consequently, there is a succinate cytoplasmic accumulation which, has been suggested that, through hypoxia‐inducible factor (HIF), leads to the creation of a beneficial microenvironment for tumor survival.^18^ The presence of a germ line mutation in the great majority of SDH deficiency cases is an indication of the syndromic nature of these neoplasias.

A relation of SDH dysfunction with renal tumorigenesis was implicated when Vanharanta et.al. reported three cases of kidney cancer, which appeared in young members of families with hereditary paraganglioma/pheochromocytoma and germline SDHB mutation.^19^ It was only after the publication of two cohort studies in 2014 and 2015^20^, ^21^ that the most recent World Health Organization classification of renal tumors accepted SDH‐deficient RCC as a special subtype of RCC with distinctive clinico‐pathological characteristics.^2^ It is a rare category of renal neoplasms with only a few case reports and case series and only two cohort studies up to date (Table 2).

**TABLE 2:** Published cases of SDH‐deficient RCC

First author, year	Cases (age*/sex)	SDH‐subunit	Detected mutations	Histology
Vanharanta S, 2004^19^	24/M, 26/M, 28/M	SDHB	R27X mutation, c.847‐50delTCTC	Clear cell carcinoma with granular‐eosinophilic cytoplasm
Ricketts C, 2008^22^	24/M,30/F,38/M, 73/M	SDHB	c.136C>T (p. Arg46Stop) in exon 2, c.137G>A(p. Arg46Gln), c.32G>A (p. Arg11His)	The 24/M and 30/F were diagnosed with ccRCC, while the 38/M with eosinophilic chromophobe RCC
Srirangalingam U, 2008^23^	16/F	SDHB	c.141G>A	Papillary RCC (type II)
Henderson A, 2009^23^	65/F	SDHB	c.600G>T	Renal oncocytoma
Housley SL,2010^24^	58/F	SDHB	2+1G ﬁ T in exon 1	RCC with giant mitochondria and features resembling both oncocytoma and chromophobe carcinoma
Gill AJ^25^	21/F, 22/M, 28/M	SDHB	c.268C>T (p. Arg90X) in exon 3, splice site mutation (c.423+1G>A) in intron 4, c.166‐170delCCTCA in exon 2	3 out of 4 tumors showed tumor cells with cytoplasmic inclusions containing eosinophilic material, while the 4^th^ case revealed features of sarcomatoid dedifferentiation
Malinoc A, 2012^26^	68/F	SDHC	c.3G>A (p.M1I), LOH of SDHC telomeric and centromeric markers: D3S3691, D3S1597, D3SVHL3, D3S1337, D3SVHL7, D3SVHL8, D3S3611	ccRCC and papillary RCC a year after the diagnosis of ccRCC
Ricketts C, 2012^27^	15/M,17/M,17/F, 19/F,25/M,27/F, 28/M,32/F,34/F, 36/M,37/M,42/F, 52/F, 55/F, 61/M,	SDHB	Exon 1 deletion, c.137G>A (p. Arg46Gln), c.268C>T (p. Arg90X), c.286+2T>A (Splice), c.379A>C (p. Ile127Leu), c.541‐2A>G (Splice), c.689G>A (pArg230His), c.286G>A (p. Gly96Ser)	Oncocytic neoplastic changes
	40/F,44/F 46/M, 49/F, 52/F, 53/F, 68/F	SDHC	c.397C>T (p. Arg133X)	ccRCC
	45/M	SDHD	c.239G>T (p. Leu80Arg)	ccRCC
Gill AJ, 2013^28^	22/F	SDHC	c.380A>G;p. His127Arg in exon 5	Neoplastic cells with eosinophilic cytoplasm and intracytoplasmic vacuoles
Papathomas TG, 2013^29^	23/Μ, 25/M	SDHB	c.3G>A (p. Met1Ile), exon 3 deletion	All RCCs displayed eosinophilic appearance and intracytoplasmic inclusions
Paik JY, 2014^29^	27/M	SDHB	c.88delC exon2 (p. Gln30A rgfsX47) in	Bubbly eosinophilic cytoplasm arranged in nests separated by a fibrovascular stroma along with eosinophilic or vacuolated cytoplasmic inclusions
MiettinenM, 2014^29^	40/M, 59/M 35/M,44/M,	N/S	N/S	ccRCC, papillary RCC, and two out of four were diagnosed as RCC of unclassified type
Gill AJ, 2014^20^	14/M,16/M,30/F, 31/F,32/F,34/M, 35/M,43/F,44/F,	SDHB	c.137G>A (p. Arg46Gln), c.725G>A (p. Arg242His), c.423+1G>A Splice, exon3deletion,c.338G>A	Focal cystic growth, uniform cytology with flocculent eosinophilic cytoplasm and intracytoplasmic inclusions
	45/M,46/M, 57/M, 76/F 54/M,		(p. Cys113Tyr),c.749C>A (p. Thr250Lys)	
WilliamsonSR,2015^21^	22/M,22/F, 32/M,40/F, 50/M,54/M, 72/M 25/M, 40/F, 54/M	SDHB	c.137G>A (p. Arg46Gln), c.859G>A (p. Arg242His), c.541‐2A>G (Splice), Exon 3 deletion	Sheets of uniform cells with oncocytic cytoplasm that contain cytoplasmic vacuoles
Jiang Q, 2015^30^	23/M	SDHA	c.2T>C (p.M1T)	Chromophobe RCC
Yakirevich E, 2015^31^	54/M	SDHA	Exon1 to 9 deletion	Mixed pattern of high grade papillary and collecting duct carcinoma with distinctive eosinophilic inclusions
Ozluk Y, 2015^32^	62/M	SDHA	splicesitedeletion (622‐2_622‐2delA)	Infiltrative pattern with solid, acinar, and papillary components; some neoplastic cells contained cytoplasmic eosinophilic inclusions
Iwashita H, 2017^32^	40/F	SDHB	c.201–2 A>C in intron 2	Tubular and solid architecture with eosinophilic granular cytoplasm and occasional vacuoles
Calió A, 2017^32^	19/M,27/F, 65/F 48/M	SDHB	c.423+1G>A,SDHBp.V140F, SDHBc.72+1G4TandTFE3 translocation	Eosinophilic cells with cytoplasmic inclusions and occasional psammoma bodies
Kumar R, 2018^33^	49/M	SDHB	N/S	Solid tumor with partially vacuolated eosinophilic cytoplasm
Li Y, 2018^34^	17/M,17/M, 20/M,21/F, 31/M, 34/M 19/M, 22/F,	SDHB loss only by IHC	N/S	Four of eight demonstrated cytoplasmic vacuoles and/or inclusions, two of eight mimicked the biphasic morphology of the t(6;11) RCC, while one was initially diagnosed as an oncocytoma
Gupta S, 2019^36^	28/M, 34/M, 65/F	SDHB loss only by IHC	N/S	Originally diagnosed as oncocytoma
Ugarte‐Camara M, 2019^36^	29/M	SDHB (retained IHC)	c.166‐170delCCTCA in exon 2	Uniform cells with eosinophilic granular cytoplasm and occasional cytoplasmic inclusions
Erickson K, 2019^37^	24/M	SDHB	N/S	Sheets of cells with clear cytoplasm, cytoplasmic inclusions and vacuoles, and areas with sarcomatoid features

SDH‐deficient RCC has, so far, been estimated to account for 0,05%‐0,2% of all RCC,^20^ presenting mainly in young adults with a mean age of 38 years (patients range from 14 to 76 years old) and a male to female ratio 1,8:1.^20^, ^21^

Histologically, they represent eosinophilic tumors with lobulated or pushing margins, occasionally surrounded, partially, by pseudocapsule and usually consisting of benign tubules or glomeruli entrapped at the borders of the neoplasm (Table 3). Solid, nested, or tubular growth patterns consisting of cuboidal to oval cells containing round nuclei with smooth nuclear membrane and dispersed chromatin without conspicuous nucleoli (neuroendocrine‐like) are typical features of SDH‐deficient RCC, but not diagnostically helpful. On the contrary, it may demonstrate overlapping features with oncocytoma or other RCC subtypes such as chromophobe or clear cell.^26^, ^31^, ^38^ The cytoplasm of these tumors has an eosinophilic or flocculent quality with vacuolation and inclusion‐like spaces containing pale eosinophilic or wispy material. Generally, they are considered low‐grade tumors but there have been described cases with ISUP nucleolar grade 3 or 4 and sarcomatoid change with or without tumoral necrosis.

**TABLE 3:** SDH‐deficient RCCs histopathologic features

SDH‐deficient RCC pathologic characteristics	
Well‐circumscribed, brown tan to red cut surface, solid (may be cystic structures)	
Solid, nested or tubular growth pattern Entrapped benign tubules	Eosinophilic cuboidal to oval cells, neuroendocrine like nuclei, cytoplasmic vacuolation, or inclusions with flocculent material
SDHB negative staining (may be also SDHA negativity)	

Immunohistochemistry (IHC) is of great importance as it is a quick, reliable, and cheap tool that can detect loss of SdhB protein expression, which is a constant feature of SDH‐deficient neoplasms, regardless of the subunit mutated. Several studies have proved the reliability of SdhB IHC in screening for syndromic disease associated with inactivation of any of the SDH subunits.^5^ Still, evaluation of SdhB staining can be tricky leading to false interpretation. More specifically, positivity is labeled with strong granular and cytoplasmic staining (same expression is observed in SDHA staining),^39^ whereas a diffuse cytoplasmic blush is considered negative. It should be noticed that without an identification of positive non‐tumoral cells (for example, endothelial cells, fibroblasts, or lymphocytes) as internal control, interpretation of staining is not accurate. On the other hand, great caution should be given at evaluating a staining as negative in tumors consisting of cells with very clear cytoplasm. Inactivation of SdhA subunit will have as a result loss of both SdhA and SdhB immunohistochemical expression. Nevertheless, immunohistochemistry's utility in detecting mutations of –C and –D subunits, respectively, has been proven to be reliable.

Differential diagnosis of SdhB‐deficient RCC includes, most commonly, other eosinophilic renal neoplasms, such as oncocytoma, eosinophilic variant of chromophobe carcinoma, hybrid oncocytic/chromophobe tumors, eosinophilic variant of clear cell RCC, and hereditary leiomyomatosis‐associated RCCs (HLRCC). Usually, the distinctive intracytoplasmic inclusions with eosinophilic flocculent material and the absence of SdhB immunohistochemical expression contribute to the diagnosis. The rare cases of SdhA‐deficient RCCs have been reported to show additionally a papillary, tubulopapillary, cribriform, and collecting duct carcinoma‐like growth pattern, and the neoplastic cells exhibit a higher nucleolar grading.^31^, ^32^ A few cases of SDHC‐ and SDHD‐deficient RCC^26^, ^27^, ^28^ have been reported which demonstrated a clear cell morphology.

On a molecular level, the most common germ line mutations of SDH‐deficient RCCs are occurring in the SDHB subunit, while mutations in SDHA, SDHC, and SDHD subunits have been only rarely detected (Table 2). It often appears in the context of an autosomal dominant tumor syndrome, including Paraganglioma pheochromocytoma, SDH‐deficient GIST, and pituitary adenoma.^2^ Although in Carney triad (paraganglioma, pulmonary chondroma, and SDH‐deficient GIST) the leading cause is hypermethylation of SDHC promoter‐specific CpG Island, such an epimutation has not been detected in SDH‐deficient RCCs.^2^ Additionally, there have not been found mutations in VHL, PIK3CA, AKT, MTOR, MET, or TP53. Recently, a study showed concurrence of TFE‐3 rearrangement and SdhB deficiency in a series of tumors.^40^ Generally, a comprehensive genetic profiling should be applied to all patients with SDH‐deficient RCCs, while first‐degree relatives should be offered a genetic counseling. Usually, SDH‐deficient RCCs are low‐grade tumors with a low metastatic risk (11%) and favorable prognosis. However, tumors with coagulative necrosis, high nuclear grade, or dedifferentiated SDH‐deficient RCC with sarcomatoid change have been described and they have a more aggressive progress and higher metastatic rate (may be up to 70%) (11). Up to date pulmonary, liver, osseous, and brain metastases have been reported.^20^, ^21^, ^27^, ^31^, ^41^

Solitary small tumors can be treated only by partial nephrectomy, while adjuvant treatment with vascular endothelial growth factor (VEGF) or tyrosine kinase inhibitors can represent the treatment of choice for patients with metastatic disease or tumors with adverse histologic features.^41^ Of great importance is the long‐term follow‐up and surveillance of these patients because of the high possibility of developing another SDH‐deficient neoplasm.^2^

## 5 CONCLUSION

In summary, SDH‐deficient RCC represents a strongly hereditary, recently described, rare entity, usually of young adulthood, with distinct clinical and pathological features. Immunohistochemistry for SDHB expression can easily confirm the diagnosis and should be performed in eosinophilic renal neoplasms, especially in young patients, or if intracytoplasmic inclusions are present. Furthermore, patients’ genetic testing and counseling, along with lifelong follow‐up would give the opportunity to them and their young family members to surveil any future incidence of the tumors linked to their germline mutation. From a clinical perspective, these tumors usually have an indolent course and should be approached in a similar fashion, with the need for lifelong surveillance to be of the utmost importance. As a result, pathologists and clinicians should keep a high index of suspicion for that kind of eosinophilic renal neoplasms.

## CONFLICT OF INTEREST

None declared.

## AUTHOR CONTRIBUTIONS

V.M. wrote the manuscript with support from D.G, D.V. and N.G. supervised the project. I.K. and E.L. helped supervise the project. N.M conducted the genetic testing and drafted the methods and genetic results section of the manuscript. J.T supervised the genetic work‐up and reviewed the methods and genetic results section of the manuscript.

## CONSENT FOR PUBLICATION

Patients signed informed consent regarding publishing their data.

## ETHICS APPROVAL

Ethical approval for this study was obtained from St. Sophia Children's Hospital Scientific and Ethics Committee (Approval number: 3669/12‐02–18).

## Data Availability

The datasets generated during and/or analyzed during the current study are not publicly available due to confidentiality agreements but are available from the corresponding author on reasonable request.

## References

[1] Moch H , Cubilla AL , Humphrey PA , Reuter VE , Ulbright TM . The 2016 WHO classification of tumours of the urinary system and male genital organs—part a: renal, penile, and testicular tumours. Eur Urol. 2016;70:93‐105. 26935559 10.1016/j.eururo.2016.02.029

[2] Tsai TH , Lee WY . Succinate dehydrogenase‐deficient renal cell carcinoma. Arch Pathol Lab Med. 2019;143:643‐647. 31026220 10.5858/arpa.2018-0024-RS

[3] Sun F , Huo Xia , Zhai Yujia , et al. Crystal structure of mitochondrial respiratory membrane protein complex II. Cell. 2005;121:1043‐1057. 15989954 10.1016/j.cell.2005.05.025

[4] McNeil MB , Clulow JS , Wilf NM , Salmond GPC , Fineran PC . SdhE is a conserved protein required for flavinylation of succinate dehydrogenase in bacteria. J Biol Chem. 2012;287:18418‐18428. 22474332 10.1074/jbc.M111.293803 PMC3365757

[5] Gill AJ . Succinate dehydrogenase (SDH)‐deficient neoplasia. Histopathology. 2018;72:106‐116. 29239034 10.1111/his.13277

[6] Gill AJ . Succinate dehydrogenase (SDH) and mitochondrial driven neoplasia. Pathology. 2012;44:285‐292. 22544211 10.1097/PAT.0b013e3283539932

[7] Desvignes JP , Bartoli Marc , Delague Valérie , et al. VarAFT: a variant annotation and filtration system for human next generation sequencing data. Nucleic Acids Res. 2018;46:W545‐W553. 29860484 10.1093/nar/gky471 PMC6030844

[8] Richards S , Aziz N , Bale S , et al. Standards and guidelines for the interpretation of sequence variants: a joint consensus recommendation of the American college of medical genetics and genomics and the association for molecular pathology. Genet Med. 2015;17:405‐424. 25741868 10.1038/gim.2015.30 PMC4544753

[9] Nozières C , Walter T , Joly M‐O , et al. A SDHB malignant paraganglioma with dramatic response to temozolomide‐capecitabine. Eur J Endocrinol. 2012;166:1107‐1111. 22430264 10.1530/EJE-11-1098

[10] Krebs HA , Johnson WA . Metabolism of ketonic acids in animal tissues. Biochem J. 1937;31:645‐660. 16746382 10.1042/bj0310645 PMC1266984

[11] Thunberg T . Studien über die Beeinflussung des Gasaustausches des überlebenden Froschmuskels durch verschiedene Stoffe. Skand Arch Physiol. 1909;22:430‐436.

[12] Renkema GH , Wortmann SB , Smeets RJ , et al. SDHA mutations causing a multisystem mitochondrial disease: novel mutations and genetic overlap with hereditary tumors. Eur J Hum Genet. 2015;23:202‐209. 24781757 10.1038/ejhg.2014.80 PMC4297908

[13] Parfait B , Chretien D , Rötig A , Marsac C , Munnich A , Rustin P . Compound heterozygous mutations in the flavoprotein gene of the respiratory chain complex II in a patient with Leigh syndrome. Hum Genet. 2000;106:236‐243. 10746566 10.1007/s004390051033

[14] Birch‐Machin MA , Taylor RW , Cochran B , Ackrell BAC , Turnbull DM . Late‐onset optic atrophy, ataxia, and myopathy associated with a mutation of a complex II gene. Ann Neurol. 2000;48:330‐335. 10976639

[15] Levitas A , Muhammad E , Harel G , et al. Familial neonatal isolated cardiomyopathy caused by a mutation in the flavoprotein subunit of succinate dehydrogenase. Eur J Hum Genet. 2010;18:1160‐1165. 20551992 10.1038/ejhg.2010.83 PMC2987458

[16] Alston CL , Davison JE , Meloni F , et al. Recessive germline SDHA and SDHB mutations causing leukodystrophy and isolated mitochondrial complex II deficiency. J Med Genet. 2012;49:569‐577. 22972948 10.1136/jmedgenet-2012-101146 PMC3500770

[17] Jackson CB , Nuoffer J‐M , Hahn D , et al. Mutations in SDHD lead to autosomal recessive encephalomyopathy and isolated mitochondrial complex II deficiency. J Med Genet. 2014;51:170‐175. 24367056 10.1136/jmedgenet-2013-101932

[18] Barletta JA , Hornick JL . Succinate dehydrogenase‐deficient tumors: diagnostic advances and clinical implications. Adv Anat Pathol. 2012;19:193‐203. 22692282 10.1097/PAP.0b013e31825c6bc6

[19] Vanharanta S , Buchta M , McWhinney SR , et al. Early‐Onset renal cell carcinoma as a novel extraparaganglial component of SDHB‐associated heritable paraganglioma. Am J Hum Genet. 2004;74:153‐159. 14685938 10.1086/381054 PMC1181902

[20] Gill AJ , Hes O , Papathomas T , et al. Succinate dehydrogenase (SDH)‐deficient renal carcinoma: a morphologically distinct entity: a clinicopathologic series of 36 tumors from 27 patients. Am J Surg Pathol. 2014;38:1588‐1602. 25025441 10.1097/PAS.0000000000000292 PMC4229399

[21] Williamson SR , Eble JN , Amin MB , et al. Succinate dehydrogenase‐deficient renal cell carcinoma: detailed characterization of 11 tumors defining a unique subtype of renal cell carcinoma. Mod Pathol. 2015;28:80‐94. 25034258 10.1038/modpathol.2014.86

[22] Ricketts C , Woodward ER , Killick P , et al. Germline SDHB mutations and familial renal cell carcinoma. J Natl Cancer Inst. 2008;100:1260‐1262. 18728283 10.1093/jnci/djn254

[23] Srirangalingam U , Walker L , Khoo B , et al. Clinical manifestations of familial paraganglioma and phaeochromocytomas in succinate dehydrogenase B (SDH‐B) gene mutation carriers. Clin Endocrinol. 2008;69:587‐596. 10.1111/j.1365-2265.2008.03274.x 18419787

[24] Housley SL , Lindsay RS , Young B , et al. Renal carcinoma with giant mitochondria associated with germ‐line mutation and somatic loss of the succinate dehydrogenase B gene. Histopathology. 2010;56:405‐408. 20459544 10.1111/j.1365-2559.2010.03482.x

[25] Gill AJ , Pachter NS , Chou A , et al. Renal tumors associated with germline SDHB mutation show distinctive morphology. Am J Surg Pathol. 2011;35:1578‐1585. 21934479 10.1097/PAS.0b013e318227e7f4

[26] Malinoc A , Sullivan M , Wiech T , et al. Biallelic inactivation of the SDHC gene in renal carcinoma associated with paraganglioma syndrome type 3. Endocr Relat Cancer. 2012;19:283‐290. 22351710 10.1530/ERC-11-0324

[27] Ricketts CJ , Shuch B , Vocke CD , et al. Succinate dehydrogenase kidney cancer: an aggressive example of the Warburg effect in cancer. J Urol. 2012;188:2063‐2071. 23083876 10.1016/j.juro.2012.08.030 PMC3856891

[28] Gill AJ , Lipton L , Taylor J , et al. Germline SDHC mutation presenting as recurrent SDH deficient GIST and renal carcinoma. Pathology. 2013;45:689‐691. 24150194 10.1097/PAT.0000000000000018

[29] Papathomas TG , Gaal J , Corssmit EPM , et al. Non‐pheochromocytoma (PCC)/paraganglioma (PGL) tumors in patients with succinate dehydrogenase‐related PCC‐PGL syndromes: a clinicopathological and molecular analysis. Eur J Endocrinol. 2014;170:1‐12. 24096523 10.1530/EJE-13-0623

[30] Jiang Q , Zhang Y , Zhou Y‐H , et al. A novel germline mutation in SDHA identified in a rare case of gastrointestinal stromal tumor complicated with renal cell carcinoma. Int J Clin Exp Pathol. 2015;8:12188‐12197. 26722403 PMC4680348

[31] Yakirevich E , et al. A novel SDHA‐deficient renal cell carcinoma revealed by comprehensive genomic profiling. Am J Surg Pathol. 2015;39:858‐863. 25724004 10.1097/PAS.0000000000000403

[32] Ozluk Y , Taheri D , Matoso A , et al. Renal carcinoma associated with a novel succinate dehydrogenase a mutation: a case report and review of literature of a rare subtype of renal carcinoma. Hum Pathol. 2015;46:1951‐1955. 26476567 10.1016/j.humpath.2015.07.027

[33] Kumar R , Bonert M , Naqvi A , Zbuk K , Kapoor A . SDH‐deficient renal cell carcinoma–Clinical, pathologic and genetic correlates: a case report. BMC Urol. 2018;18:109. 30482207 10.1186/s12894-018-0422-8 PMC6258450

[34] Li Y , Reuter VE , Matoso A , Netto GJ , Epstein JI , Argani P . Re‐evaluation of 33 ‘unclassified’ eosinophilic renal cell carcinomas in young patients. Histopathology. 2018;72:588‐600. 28898443 10.1111/his.13395 PMC7582203

[35] Gupta S , Swanson AA , Chen Y‐B , et al. Incidence of succinate dehydrogenase and fumarate hydratase–deficient renal cell carcinoma based on immunohistochemical screening with SDHA/SDHB and FH/2SC. Hum Pathol. 2019;91:114‐122. 31299266 10.1016/j.humpath.2019.07.004 PMC7528421

[36] Ugarte‐Camara M , Fernandez‐Prado R , Lorda I , et al. Positive/retained SDHB immunostaining in renal cell carcinomas associated to germline SDHB‐deficiency: case report. Diagn Pathol. 2019;14:1‐6. 31092265 10.1186/s13000-019-0812-6 PMC6521540

[37] Erickson K , Lynch D . Succinate dehydrogenase‐deficient renal cell carcinoma with sarcomatoid features. Am J Clin Pathol. 2019;152:S55‐S56.

[38] Miettinen M , Sarlomo‐Rikala Maarit , McCue Peter , et al. Mapping of succinate dehydrogenase losses in 2258 epithelial neoplasms. Appl Immunohistochem Mol Morphol. 2014;22:31‐36. 23531856 10.1097/PAI.0b013e31828bfdd3 PMC3714362

[39] van Nederveen FH , Gaal J , Favier J , et al. An immunohistochemical procedure to detect patients with paraganglioma and phaeochromocytoma with germline SDHB, SDHC, or SDHD gene mutations: a retrospective and prospective analysis. Lancet Oncol. 2009;10:764‐771. 19576851 10.1016/S1470-2045(09)70164-0 PMC4718191

[40] Calió A , Grignon DJ , Stohr BA , Williamson SR , Eble JN , Cheng L . Renal cell carcinoma with TFE3 translocation and succinate dehydrogenase B mutation. Mod Pathol. 2017;30:407‐415. 27910947 10.1038/modpathol.2016.200

[41] Paik JY , Clifton‐Bligh RJ , Gill AJ , et al. Renal carcinoma associated with succinate dehydrogenase B mutation: a new and unique subtype of renal carcinoma. J Clin Oncol. 2014;32(6):e10. 24395865 10.1200/JCO.2012.47.2647

